# The Clinical Performance of Cell Population Data for Diagnosis of Bloodstream Infection in Cancer Patients

**DOI:** 10.7759/cureus.50857

**Published:** 2023-12-20

**Authors:** Masanori Aoki, Noriyuki Watanabe, Yoshitada Taji, Yasuhiro Ebihara

**Affiliations:** 1 Clinical Laboratory, Saitama Medical University International Medical Center, Hidaka, JPN; 2 Laboratory Medicine, Saitama Medical University International Medical Center, Hidaka, JPN

**Keywords:** ly-wy, ne-sfl, mo-x, bloodstream infection, cell population data, cancer

## Abstract

Background

Bloodstream infection (BSI) induces a change in the number and morphology of blood cells. In this study, we compared cell population data (CPD) parameters between cancer patients with or without BSI to determine whether these parameters could serve as biomarkers of BSI.

Methods

Between April and June 2021, 43 BSI-negative and 22 BSI-positive cancer patients were enrolled in this study. We compared 18 CPD parameters and biomarkers between cancer patients with BSI-positive and BSI-negative.

Results

There were significant differences in the levels of several CPD parameters, including MO-WZ (p=0.040), MO-X (p<0.01), MO-Y (p=0.012), NE-SFL (p<0.01), and NE-WX (p=0.037), but not C-reactive protein (p=0.347) and procalcitonin (p=0.237) between BSI-positive and BSI-negative patients. The areas under the receiver-operating characteristic curves (AUCs) were above 0.7 for MO-X (0.762; 95% confidence intervals (CI): 0.624-0.901), NE-SFL (0.766; 95% CI: 0.625-0.880).

And LY-WY (p=0.024) showed a significant difference between gram-negative and gram-positive BSI patients with high AUC (0.883; 95% CI: 0.703-1).

Conclusion

CPD parameters (MO-X and NE-SFL) provide additional information for discriminating between BSI-negative and BSI-positive BSI. And LY-WY provides useful information for discriminating between cancer patients with gram-negative BSI and gram-positive BSI.

## Introduction

Bloodstream infection (BSI) is a leading cause of mortality worldwide. Cancer itself is associated with an increased risk of acquiring BSIs. BSI in cancer patients is one of the critical problems and sometimes influences their cancer treatments or prognosis. The various biomarkers for BSI have been investigated in attempts to make a more rapid diagnosis and achieve better outcomes. C-reactive protein (CRP) and procalcitonin (PCT) are well-known biomarkers of infection [1-3]. PCT is also a biomarker of BSI [4], and a PCT level of >1 ng/mL is strongly suggestive of bacteremia and sepsis [5].

Cell population data (CPD) parameters, which are research parameters, are measured by XN Sysmex analyzers (Sysmex Inc., Kobe, Japan) and provide values proportional to the internal complexity (x-axis), nucleic acid content (y-axis), and cell size (z-axis) of lymphocytes, monocytes, and neutrophils, respectively [6]. A severe infection like BSI causes a change in the number and morphology of blood cells, especially neutrophils and monocytes.

There have been several recent reports suggesting that CPD parameters provide useful information for the diagnosis of BSI [6-9]. However, the evaluation of CPD parameters for diagnosis of BSI in cancer patients has not yet been determined using XN Sysmex analyzers.

In this study, we compared CPD parameters between BSI-positive and BSI-negative patients with cancer to determine if these parameters could serve as biomarkers for BSI.

## Materials and methods

This retrospective single-center analysis was performed at Saitama Medical University International Medical Center. This study was approved by the Saitama Medical University International Medical Center Institutional Review Board (#2021-096) and conformed with the Declaration of Helsinki. The need for informed consent was waived in view of the retrospective nature of the study and the anonymity of the data.

Study design

A total of 65 patients who were treated between April 2021 and June 2021 and fulfilled the study eligibility criteria were enrolled in the study. All data and clinical diagnoses were obtained from the patient records. All enrolled patients were suspected to have BSI based on their clinical symptoms and laboratory data. The inclusion criteria were as follows: (1) fever suspicious for BSI and blood culture samples taken; (2) age older than 18 years; (3) no hematologic disease; (4) no admission to the ICU or high care unit during the study period; and (5) the interval between blood culture taken and the last antibiotics administration was more than three days. Patients whose microorganisms in blood culture were identified as contaminated by clinicians according to the report by Weinstein et al. were excluded [10]. All blood samples used in this study were collected within four hours from the onset of fever. Finally, BSI was diagnosed according to the criteria reported based on the clinical course [10]. Fever was defined as an axillary temperature of ≥37.5°C based on a single measurement.

Blood culture

The blood samples were collected in blood culture bottles (BACTEC® Plus Aerobic/F, Anaerobic/F; BD Biosciences, Franklin Lakes, USA). Blood culture samples were incubated using the BACTEC FX system (BD Biosciences, Franklin Lakes, USA). When the blood culture bottles were flagged positive, a subculture was made following Gram staining. When the growth of a microorganism was detected on subculture, the species was identified by matrix-assisted laser desorption ionization time-of-flight mass spectrometry on a MicroFlex LT platform (MALDI Biotyper Ver. 8.0; Bruker Daltonics, Bremen, Germany) according to the manufacturer’s instructions. The mass spectra were analyzed using Bruker Biotyper 3.4 software and library (7854 isolates; Bruker Daltonics, Bremen, Germany).

Measurement of biomarkers

Serum PCT concentrations were measured using an Elecsys BRAHMA PCT electrochemiluminescence immunoassay (Roche Diagnostics, Tokyo, Japan) with a Cobas 8000 analyzer (Roche Diagnostics, Tokyo, Japan). The measurement range is 0.02-100 ng/mL. Serum CRP concentrations were evaluated by latex-enhanced turbidimetric immunoassay using CRP-LATEX (II) X2 “SEIKEN” (Denka Seiken Co., Ltd., Tokyo, Japan) with a LABOSPECT 008 alpha automated analyzer (Hitachi High-Tech Co., Tokyo, Japan). The limit of detection was 0.010 mg/dL.

Measurement of CPD

Blood samples were collected in K2-EDTA evacuated plastic tubes (NIPRO Corporation, Osaka, Japan) and CPD was analyzed on the Sysmex XN-9100 (Kobe, Japan) within four hours from the onset of fever. We evaluated CPD parameters associated with neutrophils (NE), monocytes (MO), and lymphocytes (LY), which provide information about cell complexity, fluorescence intensity, cell size, and width of the distribution of each cell measured on the three axes of the white blood count (WBC) differential fluorescence channel.

Statistical analysis

Continuous variables are expressed as the median (interquartile range [IQR]). The Kolmogorov-Smirnov test was used to determine the normality of the distribution of continuous variables. Continuous variables were compared using the Mann-Whitney test. Fisher's exact test was used to compare categorical variables. The diagnostic properties of biomarkers and CPD parameters were evaluated by receiver-operating characteristic (ROC) curve analysis. This statistical method provides an index of diagnostic accuracy of a test as the area under the ROC curve (AUC). All statistical analyses were performed with EZR (Saitama Medical Center, Jichi Medical University, Saitama, Japan), which is a graphical user interface for R (The R Foundation for Statistical Computing, Vienna, Austria [11]. A p-value <0.05 was considered statistically significant.

## Results

The demographic and clinical characteristics of the patients are shown in Table 1. Median age was 71 years (IQR 65-77) and 39 patients were male (60.0%). None of the patients had neutropenia. Twenty-two patients were found to have BSI, and the causative pathogen was identified in each case. Three of the BSI-positive patients had fungemia.

**Table 1:** Characteristics of patients BSI-: bloodstream infection negative; BSI+: bloodstream infection positive *Values are shown as the median (IQR: Interquartile range).

Parameter	Total	BSI-	BSI+	P value
Patients, n	65	43	22	-
Male/Female, n	39/26	23/20	16/6	0.183
Median age, years (IQR)*	71 (65-77)	68 (62-77)	72 (69-75)	0.335
Disease: cancer	-	-	-	0.286
Gastroenterology, n	23	14	9	-
Respiratory, n	11	10	1	-
Gynecology, n	9	6	3	-
Urology, n	7	3	4	-
Other, n	15	10	5	-

Analysis of biomarker levels and CPD parameters between BSI-positive and BSI-negative in cancer patients

There were no significant differences in characteristics between the BSI-positive patients and the BSI-negative patients (Table 1).

We compared biomarker levels and CPD parameters between these two groups of patients. As shown in Table 2, there were significant differences between the BSI-positive and BSI-negative patients in several CPD parameters, MO-WY (p=0.025), MO-WZ (p=0.040), MO-X (p<0.01), MO-Y (p=0.012), NE-SFL (p<0.01), and NE-WX (p=0.037).

**Table 2:** The comparison of CPD between BSI negative and BSI positive groups in the patients with cancer. BSI-: bloodstream infection negative; BSI+: bloodstream infection positive; CRP: C-reactive protein, LY-X: lymphocyte complexity; LY-Y: lymphocyte fluorescence; LY-Z: lymphocyte size; LY-WX: width of dispersion of lymphocyte complexity; LY-WY: width of dispersion of lymphocyte fluorescence; LY-WZ: width of dispersion of lymphocyte size; MO-X: monocyte complexity; MO-Y: monocyte fluorescence; MO-Z: monocyte size; MO-WX: width of dispersion of monocyte complexity; MO-WY: width of dispersion of monocyte fluorescence; MO-WZ: width of dispersion of monocyte size; NE-FSC: neutrophil complexity; NE-SFL: neutrophil fluorescence; NE-SSC: neutrophil size; NE-WX: width of dispersion of neutrophil complexity; NE-WY: width of dispersion of neutrophil fluorescence; NE-WZ: width of dispersion of neutrophil size. Values are shown as the median (IQR: Interquartile range). *Procalcitonin data were obtained from 15 BSI- patients and 4 BSI+ patients.

Parameter	BSI- (n=43)	BSI+ (n=22)	P value
CRP (mg/dL)	11.01 (5.12-16.89)	12.35 (8.88-17.01)	0.285
Procalcitonin (ng/mL)*	0.33 (0.10-1.07)	1.04 (0.63-3.75)	0.250
LY-X	82 (81-83)	83 (80-85)	0.492
LY-Y	63 (59-67)	63 (59-69)	0.895
LY-Z	63 (61-64)	63 (62-64)	0.153
LY-WX	482 (438-531)	481 (413-565)	0.895
LY-WY	896 (835-985)	855 (660-949)	0.170
LY-WZ	482 (417-533)	445 (399-541)	0.505
MO-X	123 (120-124)	127 (123-130)	0.00006
MO-Y	107 (99-113)	117 (110-121)	0.012
MO-Z	67 (65-70)	68 (67-69)	0.824
MO-WX	253 (239-275)	268 (233-298)	0.421
MO-WY	721 (650-780)	603 (307-735)	0.025
MO-WZ	597 (525-650)	529 (482-597)	0.040
NE-FSC	88 (86-91)	87 (86-90)	0.852
NE-SFL	45 (42-49)	50 (47-55)	0.000502
NE-SSC	153 (150-156)	156 (152-157)	0.067
NE-WX	322 (313-340)	308 (301-325)	0.0369
NE-WY	673 (643-711)	683 (650-807)	0.409
NE-WZ	705 (651-767)	687 (660-716)	0.256

ROC analysis was performed on CPD parameters found to differ between BSI-positive and BSI-negative patients (Table 3). The AUCs for CPD parameters that showed significant between-group differences were MO-WY (0.672), MO-WZ (0.657), MO-X (0.762), MO-Y (0.693), NE-SFL (0.766), and NE-WX (0.660), respectively.

**Table 3:** Receiver-operating characteristic curve analysis of CPD for distinguishing between BSI negative and BSI positive groups CRP: C-reactive protein; MO-X: monocyte complexity; MO-Y: monocyte fluorescence; MO-WY: width of dispersion of monocyte fluorescence; MO-WZ: width of dispersion of monocyte size; NE-SFL: neutrophil fluorescence; NE-WX: width of dispersion of neutrophil complexity Values are shown as the median (IQR: Interquartile range). * Procalcitonin data were obtained from 15 BSI- patients and 4 BSI+ patients.

Parameter	AUC (95%CI)	Cutoff value	Sensitivity (95%CI)	Specificity (95%CI)
CRP	0.582 (0.439-0.726)	11.83	0.636	0.581
Procalcitonin*	0.700 (0.421-0.979)	0.780	0.750	0.667
MO-X	0.762 (0.624-0.901)	125	0.682	0.837
MO-Y	0.693 (0.552-0.834)	118	0.500	0.860
MO-WY	0.672 (0.518-0.825)	645	0.767	0.591
MO-WZ	0.657 (0.517-0.797)	516	0.767	0.500
NE-SFL	0.766 (0.625-0.880)	46	0.955	0.581
NE-WX	0.66 (0.512-0.808)	313	0.636	0.744

However, we did not find significant differences between the BSI-positive and BSI-negative cancer patients in PCT (BSI- n=15, BSI+ n=4, p=0.237) and CRP (p=0.347), which are known biomarkers for infection [1-3], PCT has also been reported to be a biomarker of BSI [4]. ROC analysis found that the AUCs for these biomarkers were ≤0.7 (Table 3).

These results indicate that two CPD parameters (MO-X and NE-SFL) might provide additional information for discriminating between BSI-positive and BSI-negative patients.

Analysis of biomarker levels and CPD parameters between gram-negative and gram-positive BSI patients

Next, we compared the biomarker levels and CPD parameters between patients with gram-negative BSI and those with gram-positive BSI. Three of our 22 patients with BSI had fungemia. Therefore, this analysis was performed in the remaining 19 patients (15 gram-negative BSI and 4 gram-positive BSI). LY-WY (p=0.024) showed a significant difference between patients with gram-negative BSI and those with gram-positive BSI (Table 4). PCT was measured in one patient in gram-positive group. The AUCs for LY-WY at cutoff value 868 was 0.883 (95% confidence interval (CI): 0.703-1, sensitivity 0.667, specificity 1.00), indicating LY-WY provide additional information for discriminating between gram-negative BSI and gram-positive BSI.

**Table 4:** The comparison of CPD between gram-negative and positive BSI in the patients with cancer BSI-: bloodstream infection negative; BSI+: bloodstream infection positive; CRP: C-reactive protein; LY-X: lymphocyte complexity; LY-Y: lymphocyte fluorescence; LY-Z: lymphocyte size; LY-WX: width of dispersion of lymphocyte complexity; LY-WY: width of dispersion of lymphocyte fluorescence; LY-WZ: width of dispersion of lymphocyte size; MO-X: monocyte complexity; MO-Y: monocyte fluorescence; MO-Z: monocyte size; MO-WX: width of dispersion of monocyte complexity; MO-WY: width of dispersion of monocyte fluorescence; MO-WZ: width of dispersion of monocyte size; NE-FSC: neutrophil complexity; NE-SFL: neutrophil fluorescence; NE-SSC: neutrophil size; NE-WX: width of dispersion of neutrophil complexity; NE-WY: width of dispersion of neutrophil fluorescence; NE-WZ: width of dispersion of neutrophil size Values are shown as the median (IQR: Interquartile range).

Parameter	BSI- (n=66)	BSI+ (n=41)	P value
CRP (mg/dL)	12.21 (8.14-17.02)	12.40 (9.85-16.40)	1.0
LY-X	82 (80-84)	80 (79-82)	0.25
LY-Y	62 (57-65)	65 (60-70)	0.411
LY-Z	63 (62-64)	64 (62-64)	0.96
LY-WX	473 (416-546)	615 (523-705)	0.177
LY-WY	813 (656-899)	1089 (931-1227)	0.024
LY-WZ	410 (391-492)	491 (431-548)	0.23
MO-X	128 (124-131)	123 (123-125)	0.294
MO-Y	116 (107-120)	114 (108-121)	0.961
MO-Z	68 (66-70)	68 (67-68)	1.0
MO-WX	274 (211-247)	262 (252-282)	1.0
MO-WY	572 (145-740)	616 (567-665)	0.736
MO-WZ	514 (489-571)	579 (561-594)	0.161
NE-FSC	88 (86-90)	89 (88-92)	0.96
NE-SFL	49 (48-58)	49 (47-50)	0.411
NE-SSC	156 (154-157)	154 (150-157)	0.516
NE-WX	308 (300-328)	316 (311-325)	0.548
NE-WY	748 (659-884)	683 (657-699)	0.307
NE-WZ	695 (660-726)	684 (653-700)	0.881

## Discussion

We have evaluated the diagnostic performance of CPD parameters for BSI on an XN Sysmex analyzer in cancer patients with BSI. In BSI, it took from hours to days to identify the microorganisms when a blood culture was flagged positive. CPD parameters could be measured on an XN Sysmex analyzer at the same time as a routine blood test, such as a complete blood count. CPD parameters show values proportional to the internal complexity (x-axis), the nucleic acid content (y-axis), and cell size (z-axis) of lymphocytes, monocytes, and neutrophils, respectively [7]. The width of dispersion of values around the mean indicates heterogeneous signals, which are calculated according to the distribution width of the major population in each cell lineage. Given that a more rapid diagnosis of BSI could lead to better outcomes, it is important to be able to detect morphological changes in WBC in response to infection, particularly BSI, in its early phase.

In our study, there were significant differences in some CPD parameters, especially in MO-X and NE-SFL, between cancer patients who were BSI-negative and those who were BSI-positive (Table 2). When the positive predictive value (PPV) and negative predictive value (NPV) were calculated using the cutoff values of MO-X (125) and NE-SFL (46), the PPV of MO-X NE-SFL were 0.650 and 0.526, and the NPV of MO-X NE-SFL were 0.800 and 0.926, respectively. The prevalence of BSI was not high with the progress of the prevention of infectious diseases and patient care, which might reflect high NPV and lower PPV of MO-X and NE-SFL, respectively.

An increased MO-X level indicates higher monocyte complexity, which might represent the increase of activated monocytes and an increased NE-SFL level indicates an increase in the cell population relative to the amount of cellular DNA and RNA, which might represent the increase of immature neutrophils. These changes might have occurred in response to the onset of BSI. And these CPD parameters indicated that they might provide additional information for discriminating between BSI-positive and BSI-negative patients.

In sepsis, monocytes/macrophages recognize pathogen-associated molecular patterns from invasive pathogens via Toll-like receptor (TLR) as its ligand, and monocytes release various cytokines that induce mobilization of immune cells [12]. Neutrophils are mobilized from marginal pools and bone marrow in response to stimulation and constantly migrate into the inflamed/damaged area. Many immature neutrophils are then supplied to peripheral blood [13]. The NE-SFL level may increase because of the abundance of nucleic acid in these immature cells.

Activated macrophages and lymphocytes might also migrate to local sites of inflammation in addition to migration of neutrophils, which could explain the above-mentioned significant increase in mobilization of neutrophils. These events might bring about a transient decrease in the number of monocytes and lymphocytes in peripheral blood numerically and proportionally. Moreover, monocytes might be more exhausted during these processes, and the differences in MO-X, MO-Y, MO-WY, and MO-WZ levels might represent a change in monocyte activation.

And LY-WY showed a significant difference between patients with gram-negative BSI and those with gram-positive BSI (cutoff value 868, AUC=0.883). These results suggested that LY-WY might be useful for discriminating between gram-positive BSI and gram-negative BSI. Discrimination between gram-negative and gram-positive BSI CPD is important to ensure treatment with appropriate antibiotics. In this regard, the role of PCT is controversial [14,15], and the diagnostic utility of CPD parameters is not known. Pathogen-associated molecular patterns derived from bacteria are recognized and activate the TLR2 pathway in gram-positive infection and the TLR4 pathway in gram-negative infection, resulting in the production of different types of inflammatory cytokines [16]. These processes might explain the differences in the LY-WY level. Therefore, LY-WY might provide better information than PCT for discriminating between gram-negative and gram-positive BSI.

There have been some reports on the performance of CPD parameters in BSI [7-9]. The subjects in these reports were not limited to cancer patients. In these reports, MO-X and NE-SFL were suggested to provide useful information for the diagnosis of sepsis/BSI. Park et al. reported that NE-SFL had an AUC of ≥0.9 for discriminating between patients with sepsis and normal controls [17]. Despite differences in study design and reference populations, similar results were obtained in our study.

PCT has been reported to be a biomarker of BSI [4]. Therefore, we used PCT in this study as a reference to assess the performance of CPD parameters in BSI. However, PCT showed little ability to identify BSI-positive patients in this study. We measured PCT in 19 of 65 patients (16 BSI-negative and 3 BSI-positive). A PCT level of >1 ng/mL is strongly suggestive of bacteremia and sepsis [5]. Our BSI-negative group contained six patients (22.2%) without infection whose PCT level was >1 ng/mL. These patients had sustained trauma or burns or had recently undergone surgery, all of which are thought to increase the PCT level [18,19]. These factors might have influenced the results of our statistical analysis. It was reported that the levels of PCT were occasionally comparable between BSI-negative and positive cancer patients [20,21].

This study had some limitations. First, it had a retrospective design. And the number of patients enrolled was small. Although no neutropenic patients were enrolled, we know that some of the treatments the patients received might have impacted changes in WBC differentiation. Apart from the influence of infection, treatment itself may modify CPD levels. We did not compare the trends in CPD levels before and after the onset of fever or during the course of treatment for underlying diseases. Finally, PCT was measured in only one-third of the patients. More PCT data would be necessary before using PCT as a reference biomarker when evaluating the performance of CPD parameters in patients. A further large-scale prospective study might be necessary to confirm the exact ability of CPD to identify BSI.

## Conclusions

In summary, we analyzed the diagnostic utility of CPD parameters in cancer patients with suspected BSI. Our results suggest that some CPD parameters (MO-X and NE-SFL) may provide additional information for discriminating between BSI-negative and BSI-positive BSI. And LY-WY may provide useful information for discriminating between gram-negative BSI and gram-positive BSI.
